# Functionalized PDMS for regulating the triboelectric output of nanogenerators: a study of charge transfer mechanisms†

**DOI:** 10.1039/d4tc05325g

**Published:** 2025-02-26

**Authors:** Jiahao Ye, Tianhuai Xu, Liva Germane, Linards Lapcinskis, Andris Šutka, Jin-Chong Tan

**Affiliations:** a Multifunctional Materials & Composites (MMC) Laboratory, Department of Engineering Science, University of Oxford Parks Road Oxford OX1 3PJ UK jin-chong.tan@eng.ox.ac.uk; b Institute of Physics and Materials Science, Faculty of Natural Sciences and Technology, Riga Technical University 1048 Riga Latvia

## Abstract

Polydimethylsiloxane (PDMS) is one of the most widely used materials in triboelectric nanogenerators (TENGs) due to its remarkable flexibility and robustness, yet its triboelectric output often limits practical applications. In this study, we present a method for tuning the triboelectric properties of PDMS through surface functionalization using self-assembled monolayers of siloxane-based molecules. Our results demonstrate that the functionalized PDMS films exhibit distinct charge donating or withdrawing behaviours, confirmed by molecular simulations and experimental characterization. Notably, trimethylsiloxyphenylmethacrylate (TMSPMA) functionalized PDMS achieved the highest voltage of 189 ± 6 V and current output of 6.75 ± 0.26 μA, leading to a 2-fold increase in peak power density compared with the standard PDMS. Moreover, to elucidate the charge transfer mechanisms between the functionalized PDMS and indium tin oxide (ITO) electrode, nanoanalytical techniques such as nano-Fourier transform infrared spectroscopy (nano-FTIR) and Kelvin probe force microscopy (KPFM) were employed to evaluate the surface chemical and electrical properties at the local scale. This research not only enhances the understanding of polymer/metal contact electrification, but also opens avenues for optimizing TENG efficiency through targeted surface functionalization strategies.

## Abstract 2

The triboelectric performance of PDMS films can be regulated through surface functionalization of self-assembled monolayers. A combination of nano-characterization techniques reveals the underlying mechanism.

## Graphical abstract

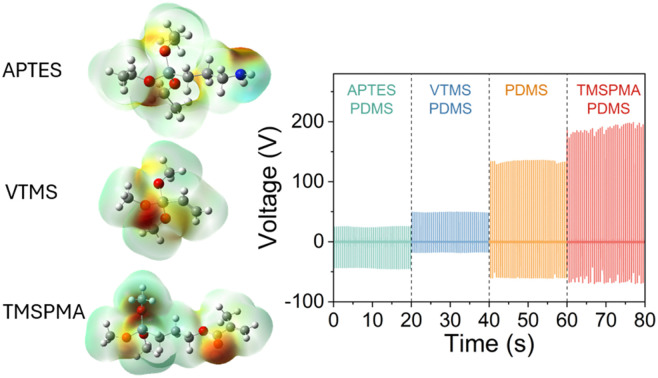

## 1. Introduction

Triboelectric nanogenerators (TENGs) are an emerging technology that converts mechanical energy into electrical energy based on contact triboelectrification and electrostatic induction. With its great advantages of scalability, simple fabrication, and flexibility, extensive research has been conducted towards commercial applications.^1,2^ Polydimethylsiloxane (PDMS), a silicone polymer with excellent mechanical resilience and electron-withdrawing ability, has become one of the most widely used polymer materials in research pertaining to TENG devices.^3^ As a soft elastomer material, PDMS exhibits several advantages during TENG device manufacturing, including ease of processing, low cost, good transparency, and potential for surface functionalization.^4,5^ Various applications of PDMS-based TENGs have been developed in biomechanical energy harvesting,^6,7^ vital sign monitoring,^8,9^ robotic sensors,^10,11^ and Internet of Things (IoT).^12^ However, the energy conversion efficiency of the prepared TENG devices is still limiting real-world applications. For example, a PDMS film generates a triboelectric output of 232 V and 6 μA while contacting with a copper electrode, as reported by Xu *et al*.^4^ To overcome the current bottleneck in the triboelectric output of TENG devices, many approaches have been made to improve the triboelectric performance of PDMS-based TENG devices including ion injection,^13,14^ surface micro-patterning design,^15,16^ and incorporating high dielectric constant fillers.^17,18^ For example, by the combination of using ion injection through an antistatic gun and surface microstructure using cilia, Seo *et al.* significantly improved the triboelectric performance of a single electrode PDMS-based TENG.^13^ In 2017, Rasel and Park fabricated microstructured PDMS using sandpaper to enlarge the contact area during triboelectrification, producing a peak-to-peak open-circuit voltage of up to 103 V against human skin.^19^ Moreover, Shi *et al.* modulated a PDMS-TENG by incorporating BaTiO_3_ and Ag nanoparticles, and improved the dielectric constant and the charge-trapping ability of the nanocomposites, yielding a maximum output voltage and current of 88 V and 8.3 μA.^20^ While these approaches contribute to performance improvements, a comprehensive understanding of the underlying charge transfer mechanism in PDMS remains elusive. The triboelectrification process involves complex surface interactions between materials, and the basic origin of the charge transfer is still being debated. The three proposed theories, including electron transfer, ion transfer, and material transfer, have been observed with experimental and computational studies under various cases,^21–25^ but a need for more refined characterization techniques is becoming evident to study these mechanisms.

Herein, we report a method to functionalize the PDMS surface with various siloxane groups to examine the effect of surface functionalization on the triboelectric output performance. Our previous findings have proven the improved charge generation properties by altering the combination of functionalized surfaces under a dielectric-to-dielectric system.^26^ In this research, we investigated the actual performance of a TENG device under a dielectric-to-metal condition by measuring the open-circuit voltage and short-circuit current output. The neat PDMS materials are prepared and then functionalized with three different types of self-assembled monolayers (SAM) with different electron-donating/withdrawing behaviour. Our results indicate that the triboelectric properties of PDMS can be tuned through surface functionalization. Moreover, using nanospectroscopy techniques, we observed variations in the work function of functionalized PDMS surfaces and evidence of bond cleavage and charged material transfer to the ITO surface, elucidating the underlying charge transfer mechanisms. The functionalization approach and discussion of mechanisms introduced in this study aimed to link nanoscale characterizations with fundamental triboelectric principles, providing guidelines to advance TENG design for higher output performance.

## 2. Methods

### 2.1 Fabrication of functionalized PDMS

Polydimethylsiloxane (PDMS) elastomer and curing agent was obtained from Dow Corning (Sylgard 184). The PDMS samples were prepared by mixing the precursor and curing agent at a mass ratio of 10 : 1. The resulting mixture was poured onto an ITO-coated glass substrate and spun at 2500 rpm for 10 seconds. After curing the PDMS samples at 80 °C for 3 h, the obtained PDMS samples were cut into squares, each with an area of 2.5 × 2.5 cm^2^ and a thickness of 100 μm. The PDMS samples were then plasma treated by oxygen to attach hydroxyl groups on the surface. Three different types of functionalized groups, (3-aminopropyl)triethoxysilane (APTES), 3-(trimethoxysilyl)propyl methacrylate (TMSPMA) and vinyltrimethoxysilane (VTMS), were dissolved in ethanol respectively at a concentration of 20 g L^−1^. The prepared PDMS was immersed in the prepared solutions for 1 h and dried in ambient air for 30 min to yield functionalized PDMS.

### 2.2 Electrical performance measurements

The triboelectric output of the as-prepared functionalized PDMS was measured under contact-separation mode. A permanent electromagnetic shaker (Brüel & Kjær LDS V201) powered by a voltage-amplified arbitrary function generator (GW Instek AFG-2105) was used to generate the periodic contact-separation motion. The ITO-contacted PDMS was then coupled with another ITO surface as the counter electrode. The TENGs were contacted for 30 minutes to reach stability. The standard electrical output was tested under impact driven by a 2 Hz square wave at a separation distance of 4 mm. The average speed of the moving electrode during contact-separation was 0.4 m s^−1^. The contact force was monitored by a load cell (RS PRO) connected to the sample holder. The instantaneous force at contact was maintained at 50 N by the controlled separation gap and shaker driving voltage. The voltage output of the samples was measured using a digital oscilloscope (PicoScope 5444B) equipped with a 100 MΩ high voltage probe (Rigol RP1300H). The current output was measured by an electrometer (Keithley 6514).

### 2.3 Material characterizations

A scanning electron microscope (SEM) was used to reveal the surface morphology of the prepared materials. The Fourier-transform infrared spectra (FTIR) were recorded by a Nicolet iS10 FTIR spectrometer equipped with an attenuated total reflectance (ATR) module. The atomic force microscopic (AFM) surface height topography and the nano-FTIR spectra *via* infrared nanospectroscopy were determined through a scattering-type scanning near-field optical microscope (Neaspec s-SNOM).^27^ The nano-FTIR spectra were taken at 20 nm resolution, combining the spectra of two laser sources with ranges from 700 to 1400 cm^−1^ and 1000 to 1600 cm^−1^. An average of 11 individual interferograms were recorded for each spectrum with a spectral resolution of 12 cm^−1^ and an integration time of 10.2 ms. The far-IR spectra (spectral range 150–650 cm^−1^) were recorded at the multimode IR imaging and microspectroscopy (MIRIAM) Beamline B22 at the Diamond Light Source synchrotron. A Bruker Vertex 80v FTIR spectrometer equipped with an ATR accessory (Bruker Optics) was used to perform the measurement. The detector was equipped with a liquid helium cooled Si-Bolometer. Post-measurement, the obtained data was processed using the OPUS 7.2 software. The Kelvin-probe force microscopy (KPFM) data were recorded using an Asylum Research Cypher ES with an ASYELEC-01-R2 conductive tip under SKPM mode. The scan area was set to 1 μm × 1 μm, with a scan resolution of 128 × 128 pixels and a scan rate of 2.44 Hz to minimize drift. The KPFM results were processed using Gwyddion software,^28^ where the color scale and range were adjusted, and the average surface potential was calculated. The pull-off test was conducted in a nanoindenter (iMicro KLA Tencor). The test employed a cylindrical flat punch with a nominal diameter of 52 μm. Initially, the flat punch was lifted 2 μm above the sample surface. Then, the tip approached the surface at a speed of 100 nm s^−1^, with surface detection facilitated by monitoring the phase signal. After contact, the punch applied a load at a rate of 0.01 mN s^−1^ until reaching a maximum load of 0.1 mN. The tip was held at this peak load for 2 seconds. Then the tip was unloaded at the same rate and retracted 5 μm from the surface. The pull-off stress was calculated by dividing the measured pull-off force by the nominal contact area. The work of adhesion was calculated by integrating the area under the load-depth curve. A total of 12 tests were performed on a 5 × 5 mm^2^ sample area.

### 2.4 Density functional theory (DFT) calculations

DFT calculations were performed to investigate the electronic properties of SAM molecules for surface functionalization. The molecular structures were first geometrically optimized utilizing the B3LYP functional combined with the 6-31G basis set^29,30^ using Gaussian 09.^31^ The electrostatic potential maps were then generated to visualize the charge distribution across the molecules.

## 3. Results

### 3.1 Materials

**Fig. 1:**
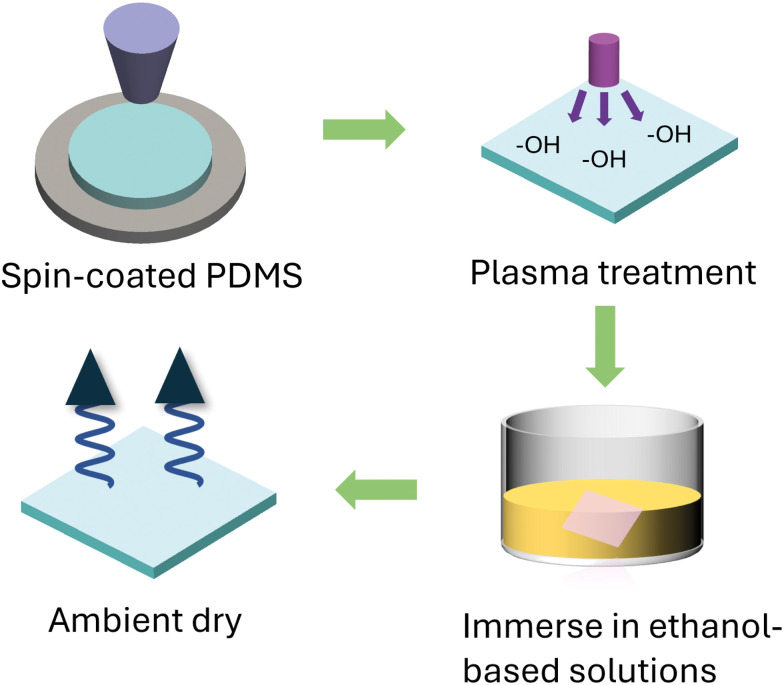
Schematic of sample preparation steps for the surface functionalized PDMS.

**Fig. 2:**
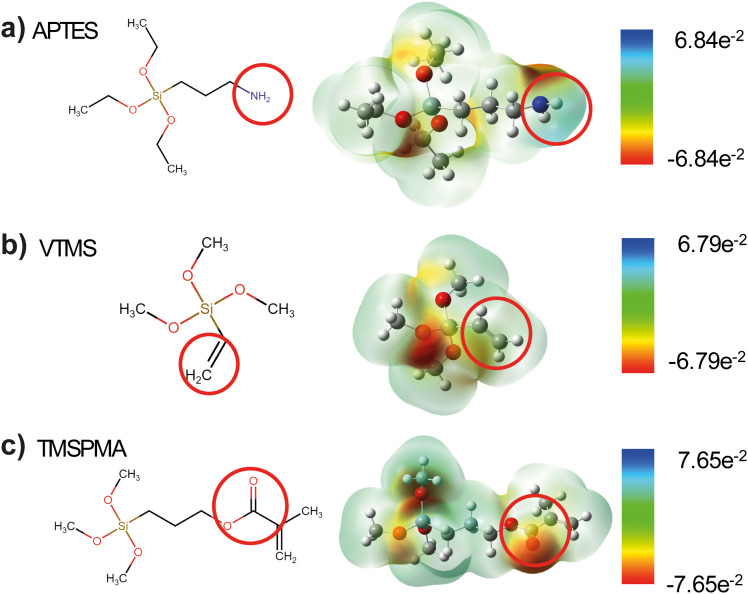
Molecular structures of the functional groups and electrostatic potential maps predicted by DFT calculations for (a) APTES, (b) VTMS, and (c) TMSPMA. Unit is kcal mol^−1^.

Fig. 1 outlines the sample preparation stages in the surface functionalization of PDMS. The pristine PDMS samples are first prepared by spin coating on ITO-coated glass followed by 3 hours of curing. The samples are then plasma treated and immersed in several siloxane-based molecules to attach certain functional groups on the PDMS surface. The surface-functionalized PDMS films show similar surface topography AFM, as shown in Fig. S1 (ESI†), with all functionalized PDMS showing surface roughness less than 1.2 nm, confirming the flatness of PDMS during contact. Fig. 2 shows the schematics of organic molecules that can be covalently linked to the PDMS backbone, including APTES, VTMS, and TMSPMA. Despite their structural similarity, these molecules were selected for their distinct functional properties. The relative electronic characteristics of these molecules are confirmed through *ab initio* density functional theory (DFT) calculations, where their electrostatic potential distributions are shown in Fig. 2. The siloxane groups in these molecules facilitate bonding with the PDMS backbone, while the distinctive parts of each molecule offer unique electronic properties to the overall structure leading to different chemical polarities. In general, the aminopropyl group in APTES offers a strong electron-donating property through the amine group within the molecule, whereas VTMS has a vinyl group that is relatively neutral due to the localized electron density around the C=C bond. On the other hand, the methacrylate bond in TMSPMA possesses a strong electron-withdrawing property resulting from the electronegative oxygen atoms in the carbonyl and ester groups, showing the most negative electrostatic potential.^32^

**Fig. 3:**
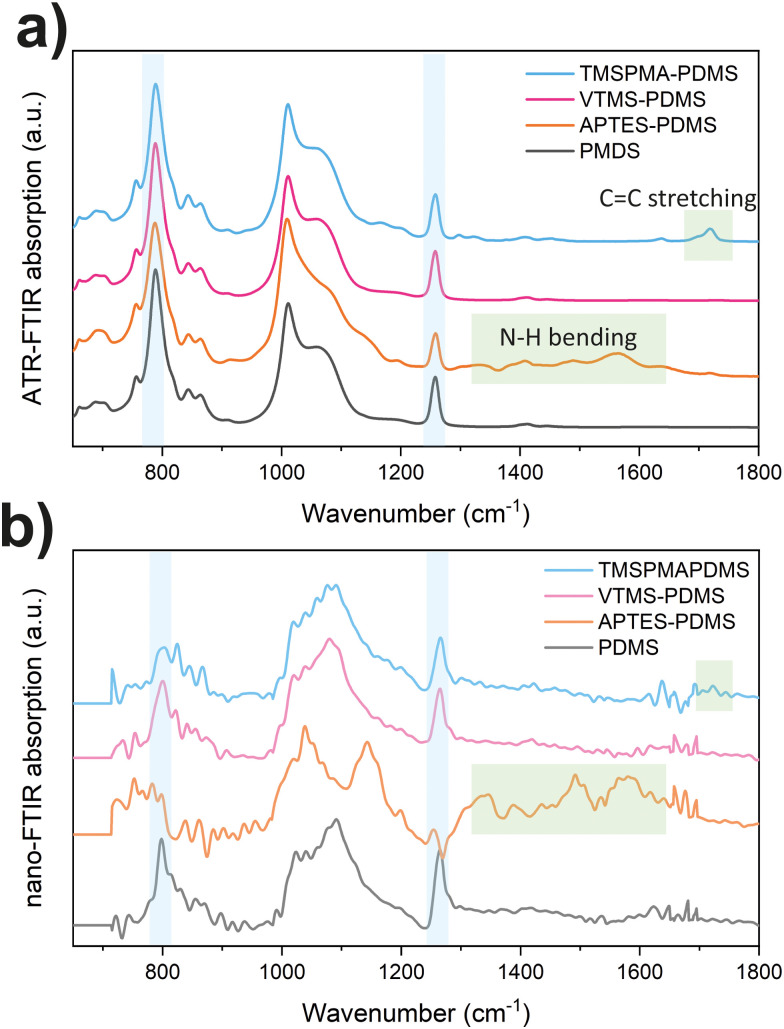
(a) ATR-FTIR and (b) nano-FTIR spectra of surface-functionalized PDMS.

The successful surface functionalization of PDMS with various functional groups has been confirmed through ATR-FTIR spectroscopy. Fig. 3a displays the mid-infrared absorption bands for the different PDMS samples. All PDMS samples show characteristic vibrational bands, including CH_3_ rocking mode at 789 cm^−1^, Si–O–Si stretching mode at 1000–1100 cm^−1^, and Si–C stretching mode at 1257 cm^−1^.^33^ In addition, the APTES functionalized PDMS shows additional bands at 1561 and 1484 cm^−1^, representing the N–H bending modes. The TMSPMA–PDMS also shows the C=O stretching mode at 1721 cm^−1^. The VTMS–PDMS displays less apparent changes in spectra, with a slight signal at 1722 cm^−1^ for C=C stretching, as shown in Fig. S2 (ESI†). The nano-FTIR spectra were also taken to confirm the obtained FTIR results, as displayed in Fig. 3b. The near-field result is well-aligned with ATR-FTIR findings from bulk samples. In the far-IR region as shown in Fig. S3 (ESI†), all functionalized PDMS exhibit similar Terahertz absorption bands, with a dominant peak observed at around 400 cm^−1^ (∼12 THz) for the Si–O–Si backbone bending and 285 cm^−1^ (∼8.5 THz) for Si–C stretching.

### 3.2 Electrical performance

**Fig. 4:**
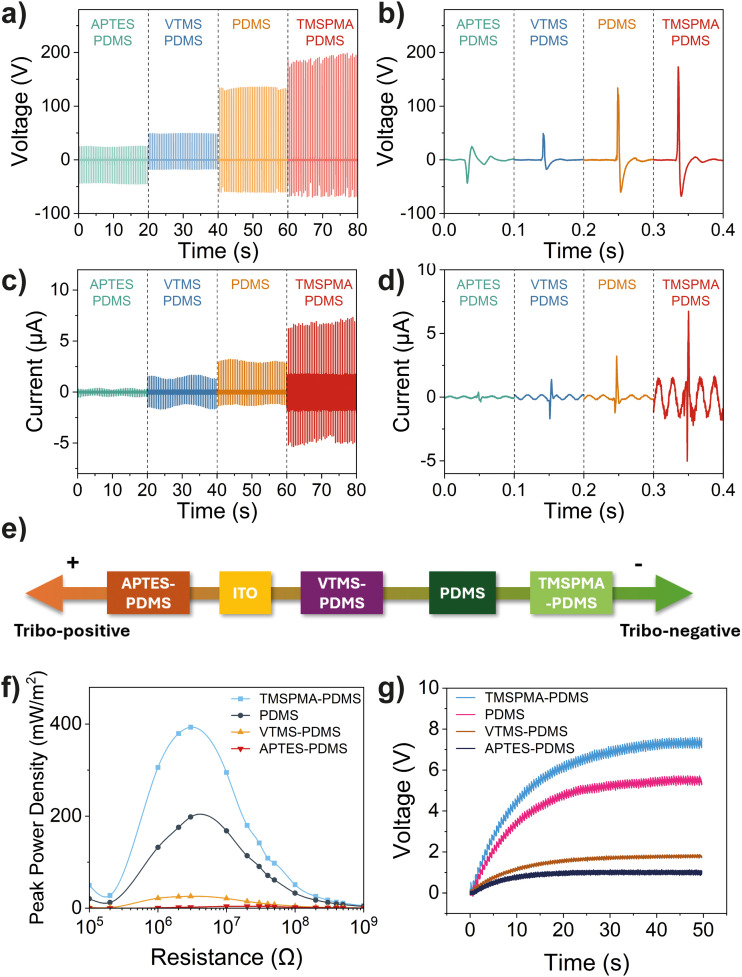
(a) Open-circuit voltage profile and (b) a single-spike voltage signal of the PDMS-based TENG. (c) Short-circuit current profile and (d) a single spike current signal of the PDMS-based TENG. (e) Triboelectric series summarizing the relative triboelectric properties of functionalized PDMS and the ITO electrode in this study. (f) Peak power densities of different PDMS-based TENGs over a range of load resistances. (g) Voltage profile across a 0.1 μF capacitor while charging by different PDMS-based TENGs.

The triboelectric outputs of the prepared PDMS samples were examined by cyclic contact-separation motion against an ITO surface, acting as the counter tribo-positive material. The experimental setup for the measurement is shown in Fig. S4 (ESI†). The maximum force at contact was maintained at 50 N for all measurements, monitored by a load cell, as shown in Fig. S5 (ESI†). Regarding the open-circuit voltage output, the standard PDMS material reaches a maximum voltage of 136 V and peak-to-peak voltage of 196 V, as shown in Fig. 4a. In contrast, APTES–PDMS and VTMS–PDMS both show reduced voltage output, with peak-to-peak voltages of only 34% that of the neat PDMS. On the other hand, TMSPMA–PDMS, functionalized with a more electronegative functional group, demonstrates a 1.42 times improvement in maximum voltage and 2.25 times improvement in maximum current, showing the highest voltage and current output of 189 ± 6 V and 6.75 ± 0.26 μA, derived from 40 contact-separation cycles. Although APTES–PDMS and VTMS–PDMS show similar voltage output in value, their output profile differs significantly. When looking at a single spike of electric signal generated during contact and separation in Fig. 4b, all PDMS materials, except for APTES–PDMS, show a positive voltage during contact followed by a negative voltage during separation. Typically, PDMS material is considered an excellent tribo-negative material, which means that it withdraws and holds negative charges on its surface in a triboelectric pair. While the PDMS and ITO surfaces are in contact, the surface of PDMS gets negatively charged by taking away the free electrons on the conductive ITO surface. However, an inverted phenomenon was observed for the case of APTES–PDMS, under the premise that the electrical connections during data collection are the same for all samples, indicating that the APTES–PDMS has become a more tribo-positive material relative to ITO. The APTES–PDMS donates electrons to ITO during contact, resulting in the reversed voltage profile exhibited in Fig. 4b. The strong electron-donating effect of the amine group in APTES resulted in changes in the original surface properties of PDMS. Although VTMS–PDMS also reduces the triboelectric output due to the neutral surface alkene groups, the reduction does not alter its relative position in the triboelectric series compared to ITO.

A similar trend is observed in the closed-circuit current output as shown in Fig. 4c. APTES–PDMS shows the lowest current output, followed by VTMS–PDMS, neat PDMS, and TMSPMA–PDMS. Although APTES–PDMS and VTMS–PDMS have similar voltage output, the current for APTES–PDMS is much lower. While the output voltage reflects the potential difference between the contacting materials, the measured current compensates the induced charges on the electrodes. As ITO is a conductive material abundant with free charge carriers, it has limited ability to hold or transfer more electrons from the APTES–PDMS side, resulting in the low current output of APTES–PDMS. When examining the individual signal peaks in Fig. 4d, APTES–PDMS has the only reversed output peak shape compared to other membranes, confirming our findings in voltage measurements. It is widely accepted that the surface property of a material plays a vital role in its triboelectric performance. While modifying the material surface with various functional groups, the work function of the material compared to the electrode is altered, leading to different charge transfer phenomena. These triboelectric performance results align with our DFT predictions, as shown in Fig. 1c, highlighting the role of surface functionalization in tuning charge transfer characteristics. Summarizing the relative electron-donating and withdrawing propensities of PDMS materials during contact, a triboelectric series can be established for materials studied in this work, as shown in Fig. 4e. Among these, APTES–PDMS is the only material that exhibits stronger electron-donating behaviour compared to conductive ITO, placing it as the most tribo-positive material in the series. On the other hand, through functionalization, TMSPMA–PDMS with enhanced withdrawing ability ranks furthest to the right within the series.

The peak power density of the prepared materials was measured and calculated by connecting the TENG devices to loads with varying resistances. The closed-circuit voltage was measured across the resistors, and the peak power density was calculated using *P*_d_ = *V*^2^/(RA). As summarized in Fig. 4f, the TMSPMA-modified PDMS exhibits the highest power density of 393 mW m^−2^, which has doubled the power output of the neat PDMS attributed to the higher voltage and current output of the former, whereas APTES–PDMS shows the lowest power output. It is worth noticing that all these materials show their highest power density at around 3 MΩ, except for APTES–PDMS, which peaks at 10 MΩ. This optimum resistance at the highest power density denotes the internal resistance of the device.^34^ The high internal impedance of the APTES–PDMS device can be explained by the high electron transfer resistance between the surfaces of two tribo-positive materials. Individual measurements of voltage-resistance relationships for functionalized PDMS are shown in Fig. S6 (ESI†). The alternating charge transferred during contact-separation can be rectified into direct current and harvested by capacitors for real-world applications. Fig. S7 (ESI†) shows the charging curves for capacitors ranging from 0.1, 0.47, 1, and 10 μF capacitances. Comparing the charging rates for a 0.1 μF capacitor, we found that TMSPMA–PDMS achieved the fastest charging speed, reaching a voltage of 7.3 V in 50 s, as shown in Fig. 4g.

### 3.3 Charge transfer mechanism

The charge transfer mechanism of the prepared PDMS-based TENGs is revealed and discussed based on results from various nano-scale characterization techniques. Among the three charge transfer mechanisms, the ion transfer model explains charge transfer by the formation of a water layer on the surface. This model normally applies to ionic and hydrophilic polymers. However, in our case, PDMS has high hydrophobicity where the water layer and amount of charge transfer through ions can be neglected since surface ionization reaction is unlikely to happen between the hydrophobic solid surfaces.^35^ We therefore mainly elucidate the role of electron transfer and mass transfer mechanisms during the mechanical interaction between functionalized PDMS and ITO surfaces.

**Fig. 5:**
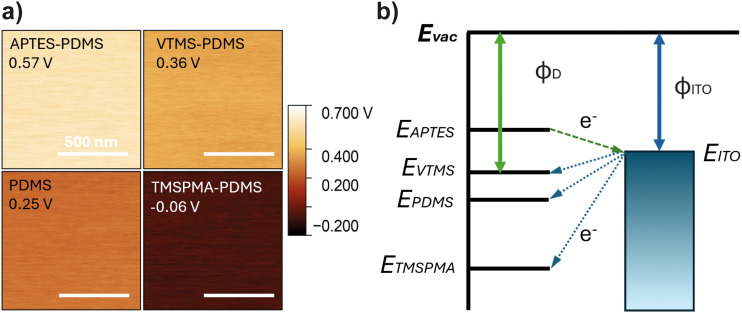
(a) KPFM surface potential maps and average *V*_CPD_ values of different functionalized PDMS after stabilization. (b) Schematic diagram of the contact electrification mechanism elucidated by the electron transfer between PDMS and ITO due to different work functions. *E*_vac_, vacuum level; *E*_X_, Fermi level of the functionalized PDMS; *ϕ*_D_, work function of functionalized PDMS; *ϕ*_ITO_, work function of the ITO electrode. The relative position of PDMS samples in the energy diagram is determined by the measured *V*_CPD_ values, where *E*_X_*= E*_vac_ − (*ϕ*_tip_ − *e*·*V*_CPD_X_).

From the electron transfer perspective, electrons are transferred from a material with lower work function to higher, and the amount of charge transfer is proportional to the difference in the work function of the two materials. In this study, the work function of functionalized PDMS (*Φ*_sample_) was indirectly measured by KPFM through the measurement of contact potential difference (*V*_CPD_):

1 *Φ*_sample_ = *Φ*_tip_ − *e*·*V*_CPD_

where *Φ*_tip_ is the work function of the KPFM probe tip and *e* is the charge amount carried by an electron. According to eqn (1), a higher measured contact potential difference corresponds to a lower surface work function. To measure the contact potential difference of PDMS samples, pristine polymers were cut into squares of 5 mm × 5 mm and stabilized for two days after sample preparation to ensure no accumulated charge on the surface. Fig. 5a demonstrates the average *V*_CPD_ values and surface potential distribution maps of functionalized PDMS samples measured under stabilized conditions. The functionalized PDMS samples exhibited a relatively homogeneous surface potential, with APTES–PDMS showing the highest *V*_CPD_ and, therefore, the lowest work function, while TMSPMA–PDMS showed the lowest *V*_CPD_ and highest work function, consistent with the trend in previously summarized triboelectric series in Fig. 4e. The work function of a material is the minimum thermodynamic energy required to remove an electron from a solid to a point just outside the solid surface.^36^ Electrons are transferred from the material with a lower work function to higher in order to maintain Fermi level balance, resulting in the material with lower work function being positively charged after contact.^37^ This behaviour is illustrated in the surface state model to explain the electron transfer phenomenon as proposed in Fig. 5b. Here, the energy levels of functionalized PDMS are ranked according to the measured *V*_CPD_ values in comparison to the counter material ITO. While the APTES–PDMS has the lowest work function, its Fermi level exceeds the energy level of ITO, thereby transferring electrons during contact. Conversely, when the PDMS surface is functionalized with TMSPMA, the work function increases, resulting in a lower Fermi level. As a result, this increases the energy gap between TMSPMA–PDMS and the ITO electrode, compared to neat PDMS, thereby driving more electrons to hop from the ITO surface to TMSPMA–PDMS and enhancing charge generation within a TENG device.

**Fig. 6:**
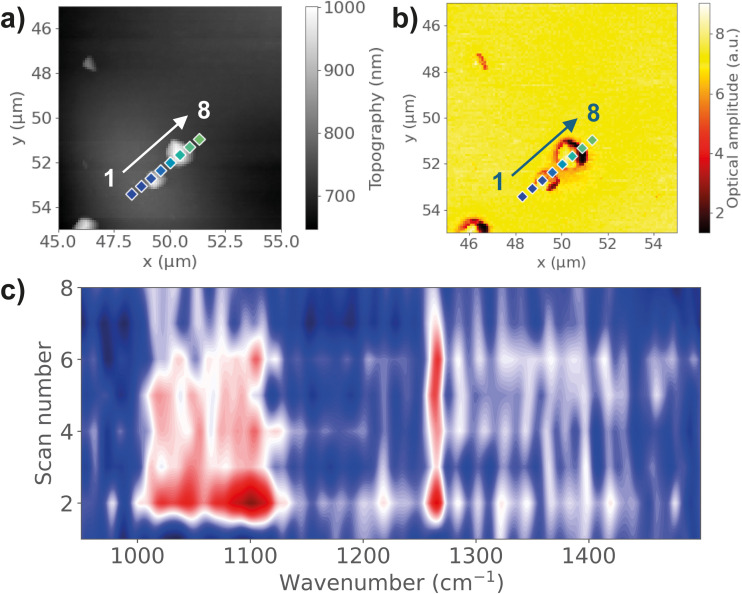
(a) AFM topography and (b) near-field IR absorption image of nanoscale PDMS residues detected on the ITO surface after contact. (c) Nano-FTIR linescan spectra corresponding to the points marked on the AFM image.

On the other hand, evidence of material transfer has been observed *via* nano-FTIR analysis and KPFM scans. The mass transfer or material transfer model proposes that the charge transfer between two materials is due to the transfer of small fragment materials that carry a certain amount of charge that adhere to another surface during contact. As a soft polymer material with relatively low Young's modulus and high surface adhesion, PDMS is more likely to transfer materials during contact from the high contact area and higher density of *van der Waals* intermolecular bonds.^38^ To examine the mass transfer between the PDMS film and ITO electrode, pristine materials were clamped together using spring clamps under 6.9 N to ensure conformal surface contact and eliminate potential contamination effects. Then the materials were separated and evaluated under AFM integrated nano-FTIR spectroscopy at a 20-nm spatial resolution.^27^ The surface topography of the contacted ITO surface from AFM shows increased surface roughness with particles of different sizes. Fig. 6a shows the observation of several nano-sized particles on the ITO surface. By scanning the nano-FTIR spectra across the particles, characteristic peaks at 1265 cm^−1^ and 1091 cm^−1^ representing Si–C stretching and Si–O–Si stretching were observed on the particles, which confirmed the presence of PDMS. The IR absorption peaks are only observable near the particles, but not on the smooth ITO surface, demonstrating the transfer of PDMS residues during contact. In addition to nano-sized PDMS particles, larger transferred fragments, up to 10 μm, are also observed, as shown in Fig. S8 (ESI†). In this case, the absorption peaks are detectable even far away from the particles, indicating a layer of PDMS being transferred to the ITO surface, not only of the large particles but also with cleavage of bonds. The same observation is found for TMSPMA–PDMS, as shown in Fig. S9 (ESI†). Comparing the relative peak intensity of 1265 cm^−1^ and 1091 cm^−1^, the Si–O–Si peak shows a lower peak intensity at the ITO surface, compared with bulk TMSPMA–PDMS. This could be an indication of Si–O bond breaking due to physical contact, where the chemical bond breakage on the backbone of functionalized PDMS leads to the transfer of small material fragments across the contact interface.^4^ These fragments carry charges due to chemical bond breaking, resulting in the transfer of charges when they come into contact with the counter material.

**Fig. 7:**
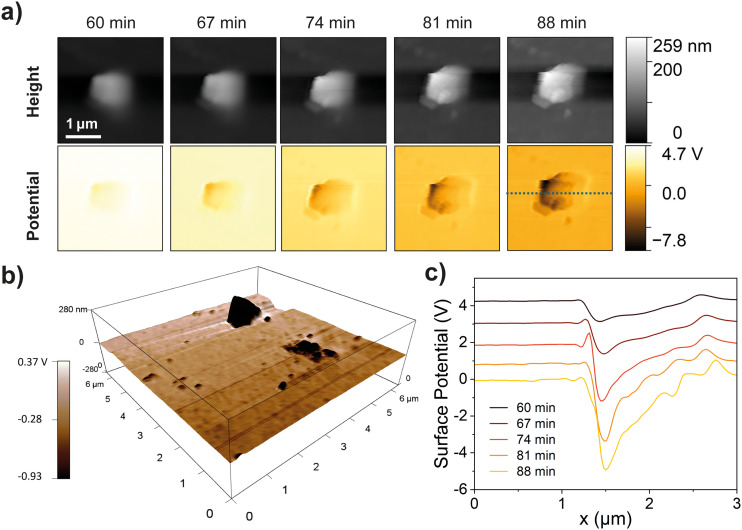
(a) AFM height topography and KPFM surface potential of a PDMS residue on the ITO surface after contact. (b) 3D maps of the height profile topography with the KPFM surface potential superimposed with corresponding colour scales of PDMS contacted ITO surface. (c) Surface potential profile across the PDMS residue on the ITO surface at different times after contact.

**Fig. 8:**
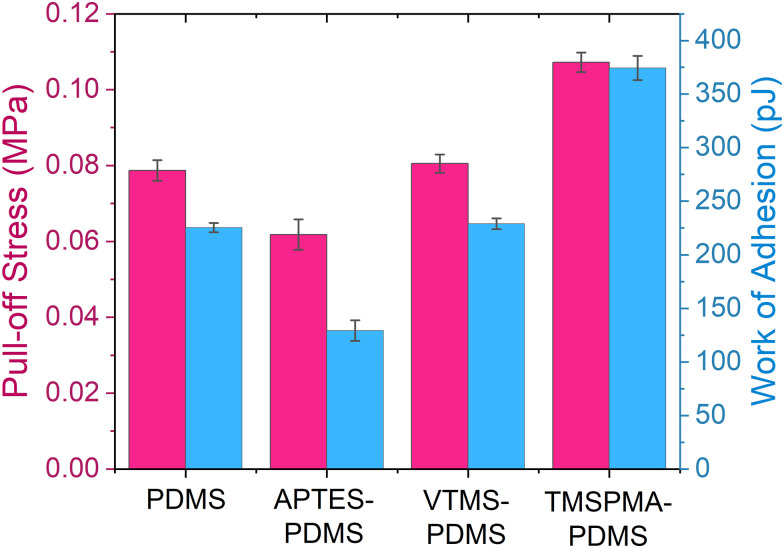
Comparison of the pull-off stress and work of adhesion for the pristine and functionalized PDMS samples. Work of adhesion values were obtained using a cylindrical flat punch with a nominal diameter of 52 μm. Mean and standard deviation values were derived from 12 measurements shown in Fig. S11 (ESI†).

The KPFM results further support the material transfer mechanism. In Fig. 7a, we detected rounded PDMS residue on the ITO surface immediately after contact, showing a significantly more negative surface potential than the pristine ITO surface. The 3D topography and potential distribution of PDMS residues revealed sizes from 2 μm down to the nanoscale, as shown in Fig. 7b. The surface charge of ITO initially after contact shows a highly positive value due to the withdrawal of electrons from the conductive surface, then gradually drops as it stabilizes and discharges under ambient conditions. However, it can be observed that the PDMS residue retains a much lower surface potential compared to the ITO surface, as shown in Fig. 7c, indicating that these charged tribo-negative counterpart fragments transferred to the surface may contribute importantly to charge transfer during contact. In addition to the observed transferred polymer fragments on the electrode surface, further experiments have revealed the correlation between the surface adhesion properties and triboelectric output of the materials, as shown in Fig. 8. Detailed experimental methods and results are presented in Fig. S10 and S11 (ESI†). TMSPMA–PDMS shows the highest pull-off stress and work of adhesion of 0.11 MPa and 374.4 pJ, respectively, relative to the other functionalized PDMS, indicating a more adhesive surface and thus a higher acceleration during separation. The high pull-off stress and work of adhesion promote more bond cleavage, leading to more material and charge transfer. The functionalization of PDMS alters the surface mechanical properties, further contributing to improved charge transfer efficiency. Together, these observations highlight the critical role of surface adhesion and material transfer in optimizing the triboelectric performance.

## 4. Conclusion

In summary, we introduced a surface functionalization method to tune the surface triboelectric properties of PDMS by self-assembled monolayers of APTES, VTMS and TMSPMA. The functionalized PDMS shows different relative positions on the triboelectric series due to the introduction of surface chemical bonds, validated through molecular simulations and experimental characterizations. The TMSPMA functionalized PDMS shows the highest voltage and current output of 189 ± 6 V and 6.75 ± 0.26 μA, increasing the peak power output density by 2 times compared with the neat PDMS. Moreover, advanced nanoanalytical techniques, including nano-FTIR and KPFM, were employed to investigate the charge transfer mechanisms between the functionalized PDMS and an ITO counter electrode. Our findings indicate that the surface functionalization approach varies the surface work function of PDMS, potentially serving as the driving force for the charge transfer against the ITO electrode. In addition, evidence of material transfer at the nanoscale is observed through surface topographical changes and chemical composition variations, which also significantly contributes to charge transfer. Future studies can leverage these insights in studying the mechanisms under polymer/metal contact electrification and improving the triboelectric performance through strategic surface modification strategies.

## Author contributions

Jiahao Ye: conceptualization, methodology, investigation, formal analysis, writing – original draft preparation, writing – review & editing. Tianhuai Xu: methodology, formal analysis, writing – review & editing. Liva Germane: methodology, investigation, funding acquisition. Linards Lapcinskis: methodology, investigation. Andris Šutka: conceptualization, methodology, supervision, funding acquisition, writing – review & editing. Jin-Chong Tan: conceptualization, methodology, supervision, funding acquisition, writing – review & editing.

## Data availability

The data supporting this article have been included as part of the ESI.†

## Conflicts of interest

The authors declare that they have no known competing financial interests or personal relationships that could have appeared to influence the work reported in this paper.

## Supplementary Material

TC-013-D4TC05325G-s001
