# Supplementary material for: Functionalized PDMS for regulating the triboelectric output of nanogenerators: a study of charge transfer mechanisms
Source: J Mater Chem C Mater. 2025 Feb 26;13(15):7654–63. doi: 10.1039/d4tc05325g (PMC11899533; doi:10.1039/d4tc05325g)
Supplement: TC-013-D4TC05325G-s001 [file TC-013-D4TC05325G-s001.pdf]

Supplementary Information (SI) for Journal of Materials Chemistry C.
This journal is © The Royal Society of Chemistry 2025

Supplemental Information

for

Functionalized PDMS for Regulating Triboelectric Output of Nanogenerators: A Study of Charge Transfer Mechanisms

*Jiahao Ye,[1] Tianhuai Xu, [1] Liva Germane,[2] Linards Lapcinskis,[2] Andris Šutka,[2] and Jin-Chong Tan[1,]**

[1] Multifunctional Materials & Composites (MMC) Laboratory, Department of Engineering Science, University of Oxford, Parks Road, Oxford OX1 3PJ, U.K.

[2] Institute of Materials and Surface Engineering, Faculty of Natural Sciences and Technology, Riga Technical University, 1048 Riga, Latvia.

*Corresponding author's e-mail: jin-chong.tan@eng.ox.ac.uk

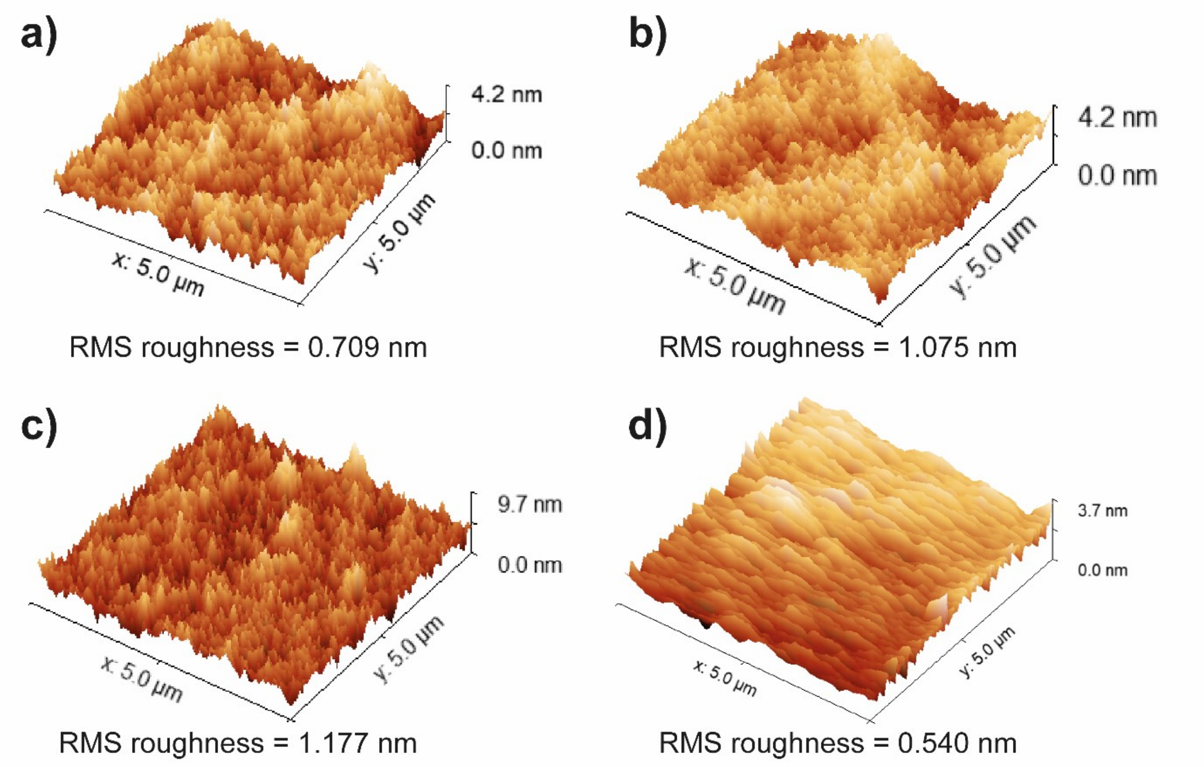

Figure S1. AFM 3D topography of a) PDMS, (b) VTMS-PDMS, (c) APTES-PDMS, and (d) TMSPMA-PDMS surfaces. The RMS roughness values were calculated using Gwyddion Software [1] employing scan areas of 5 × 5 µm.

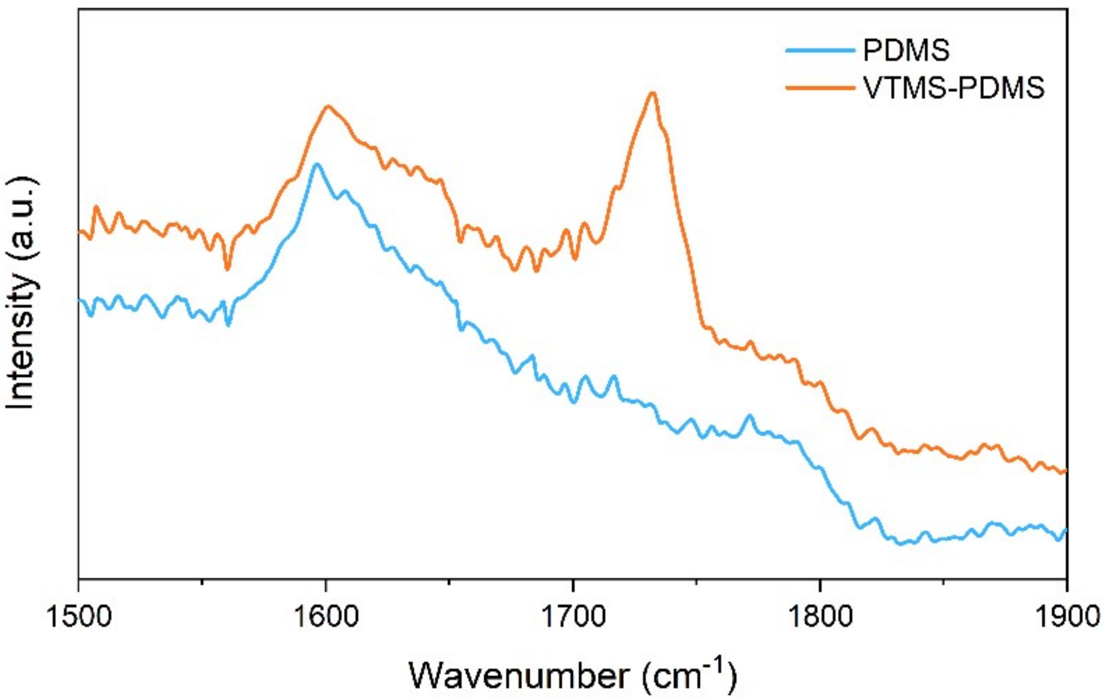

Figure S2. Comparison of ATR-FTIR spectra of PDMS and VTMS-PDMS between 1500 cm^{-1} and 1900 cm^{-1}.

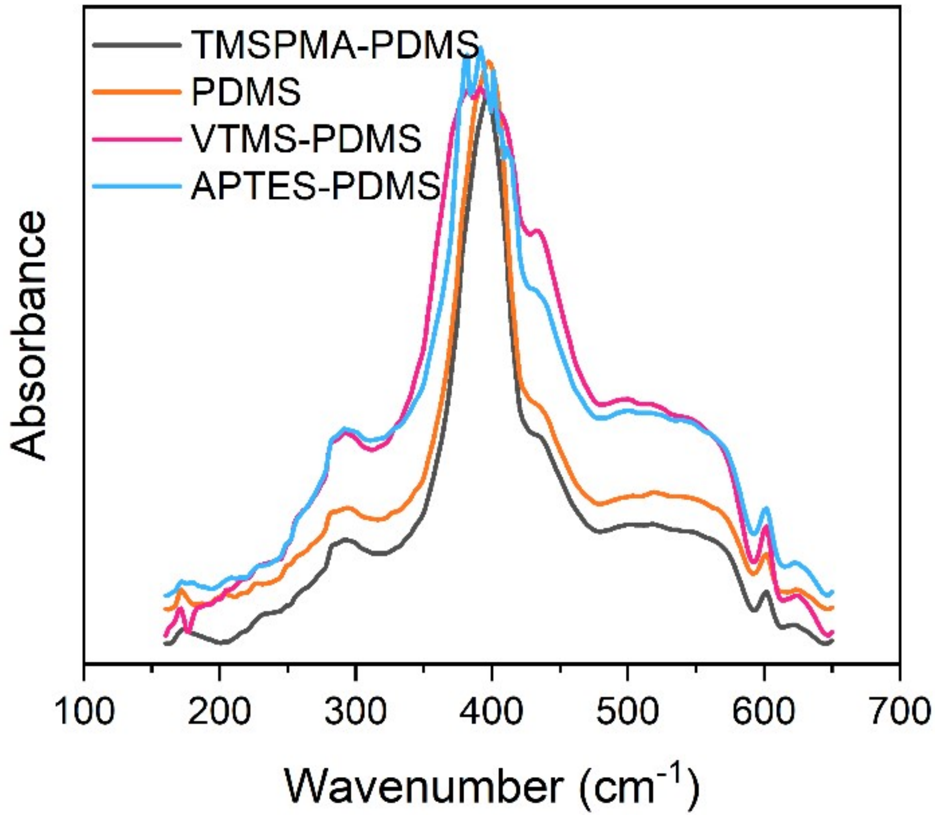

Figure S3. Synchrotron-based far-IR spectra of functionalized PDMS surfaces. Measurements were conducted at a resolution of 4 cm^{-1}, with each spectrum obtained by averaging 256 scans to ensure a high signal-to-noise ratio. Additionally, each spectrum represents the average of 10 individual measurements at different locations. The results indicate that the functionalization process did not induce detectable shifts in other chemical bond vibrations within this spectral range.

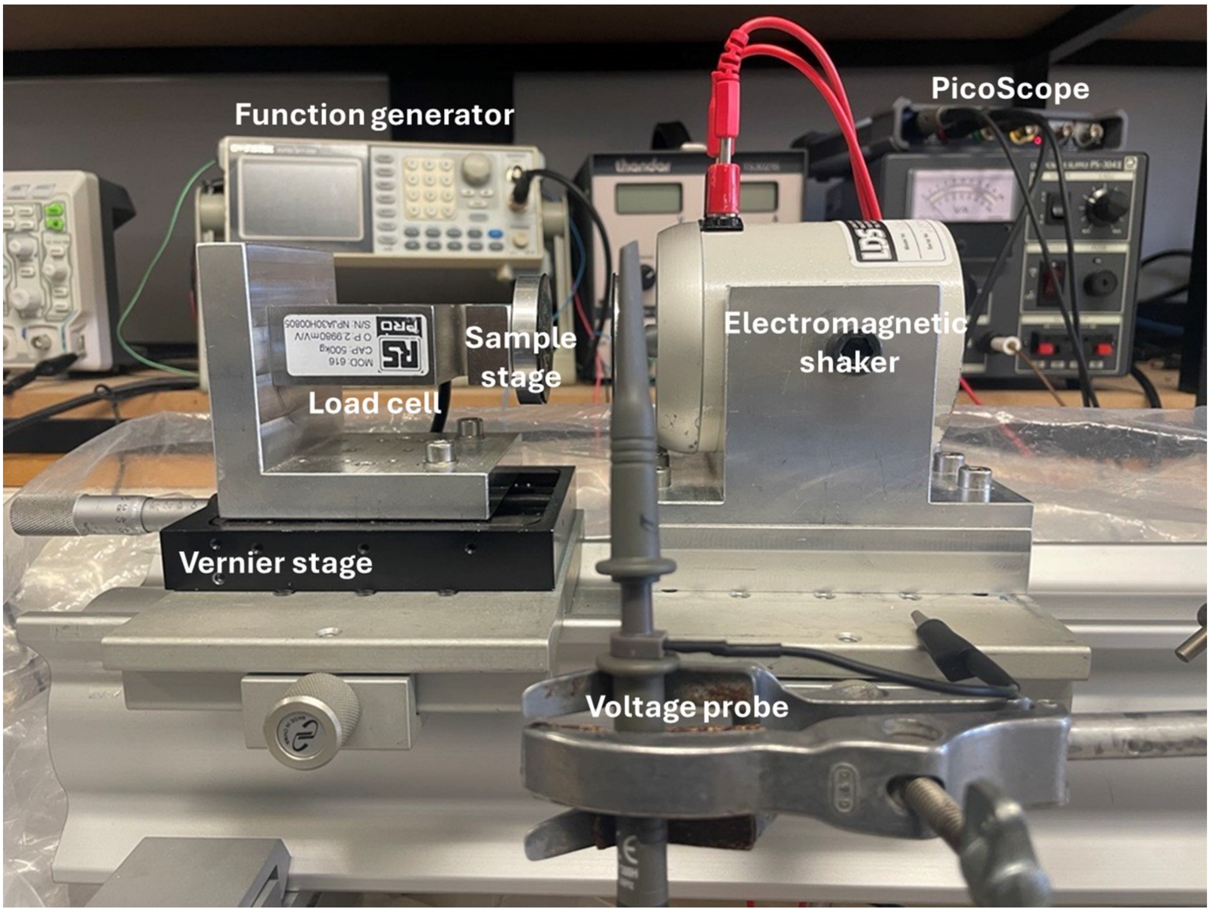

Figure S4. Experimental setup for electrical performance measurement under contact-separation mode for functionalized PDMS samples against ITO.

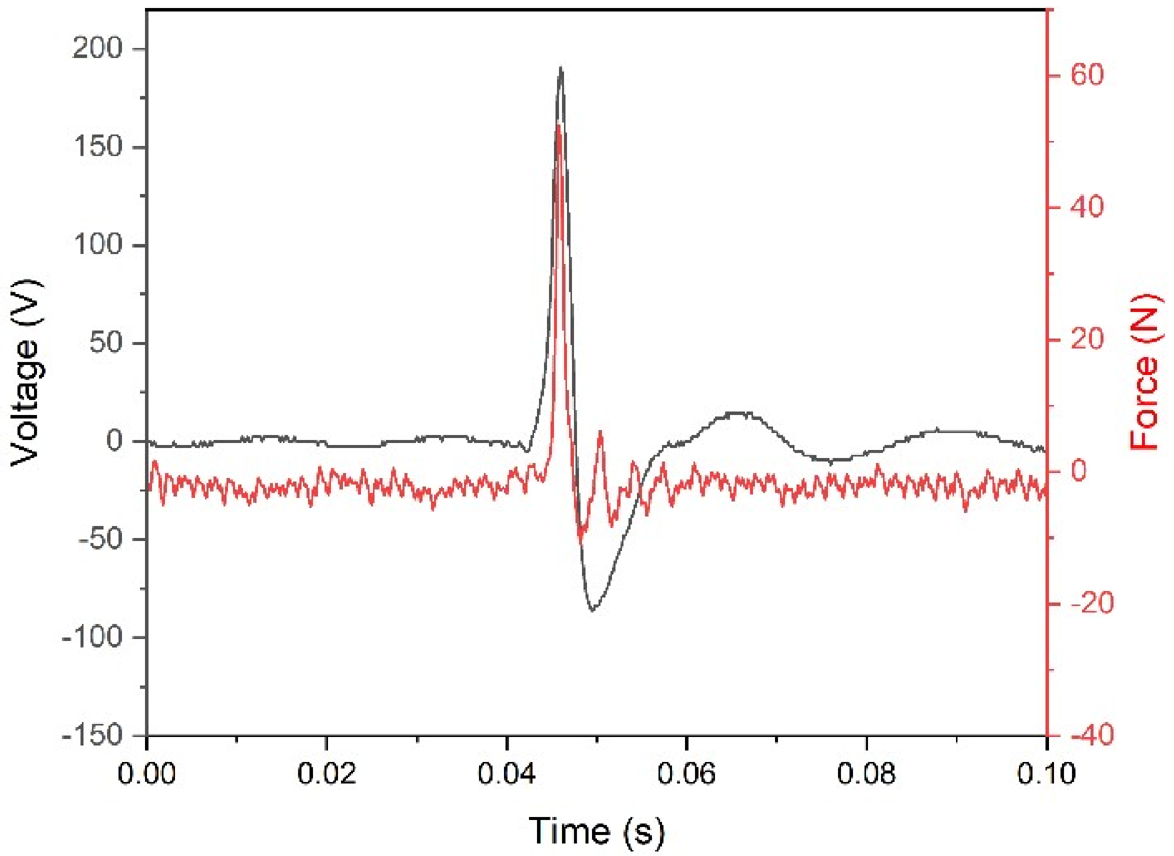

Figure S5. Correlation between the contact force and output voltage during a cycle of contact-separation process. The maximum instantaneous force is ~50 N.

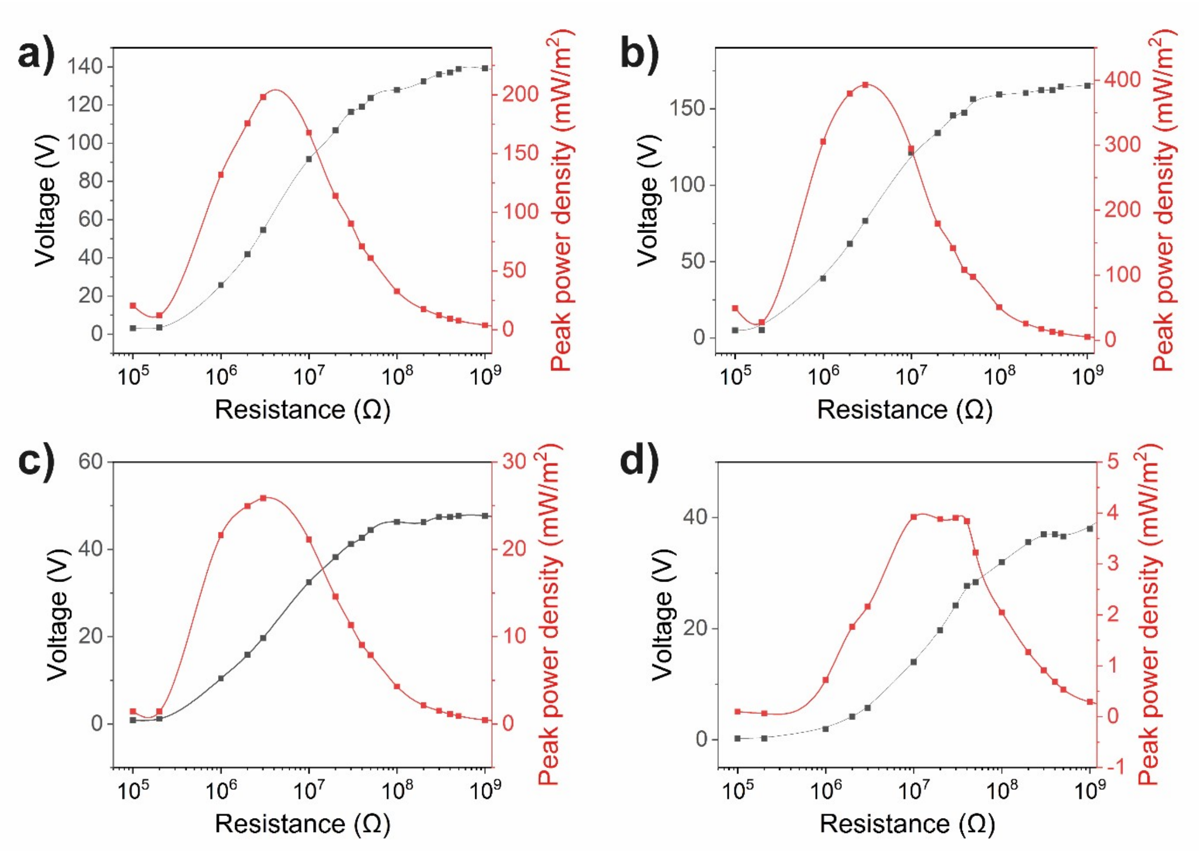

Figure S6. Variation of peak power output density and voltage output relative to different load resistances for (a) PDMS, (b) TMSPMA-PDMS, (c) VTMS-PDMS, and (d) APTES-PDMS based TENG devices. All tests conducted against the ITO surface as counter electrode.

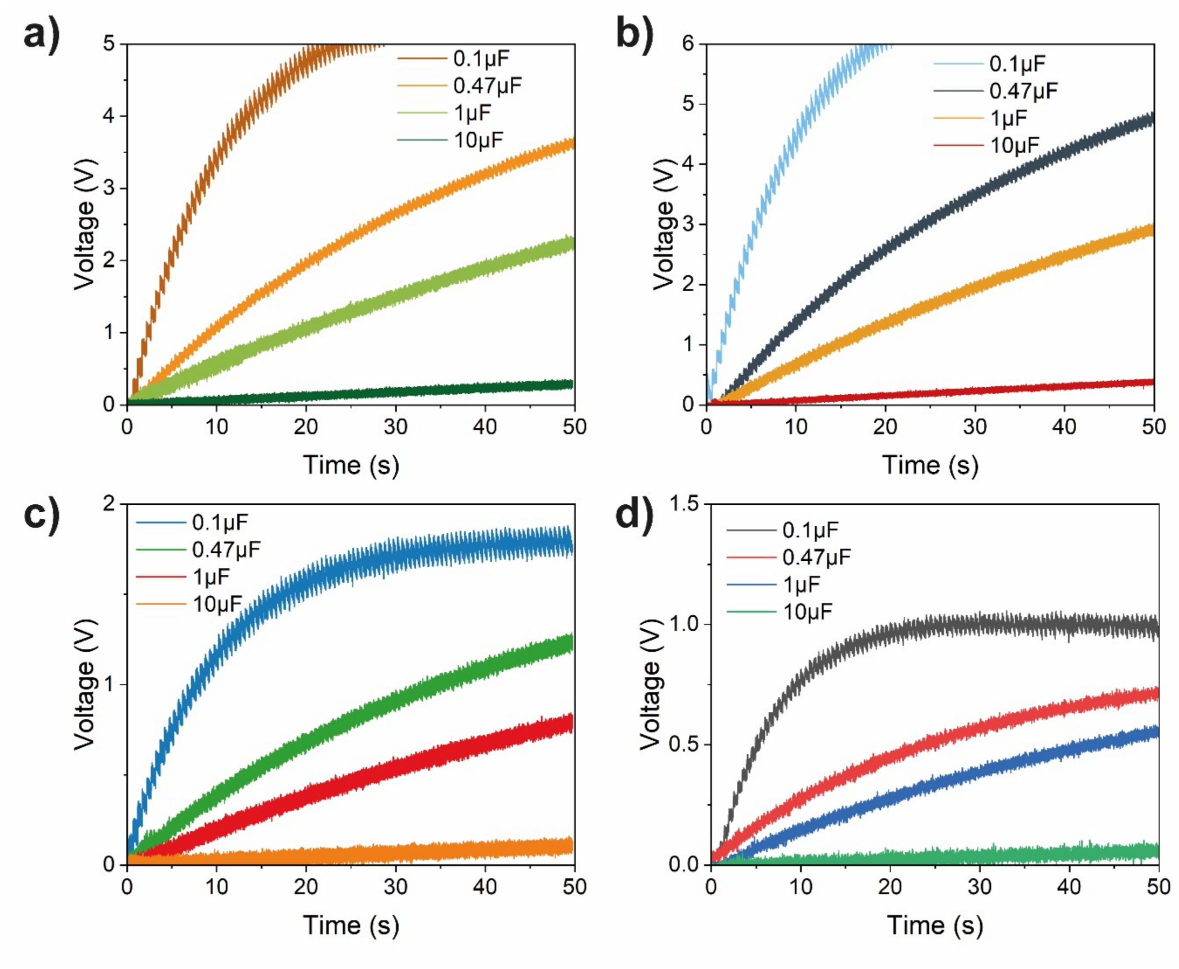

Figure S7. Voltage profiles across capacitors with different capacitances when charged using (a) PDMS, (b) TMSPMA-PDMS, (c) VTMS-PDMS, and (d) APTES-PDMS based TENG devices.

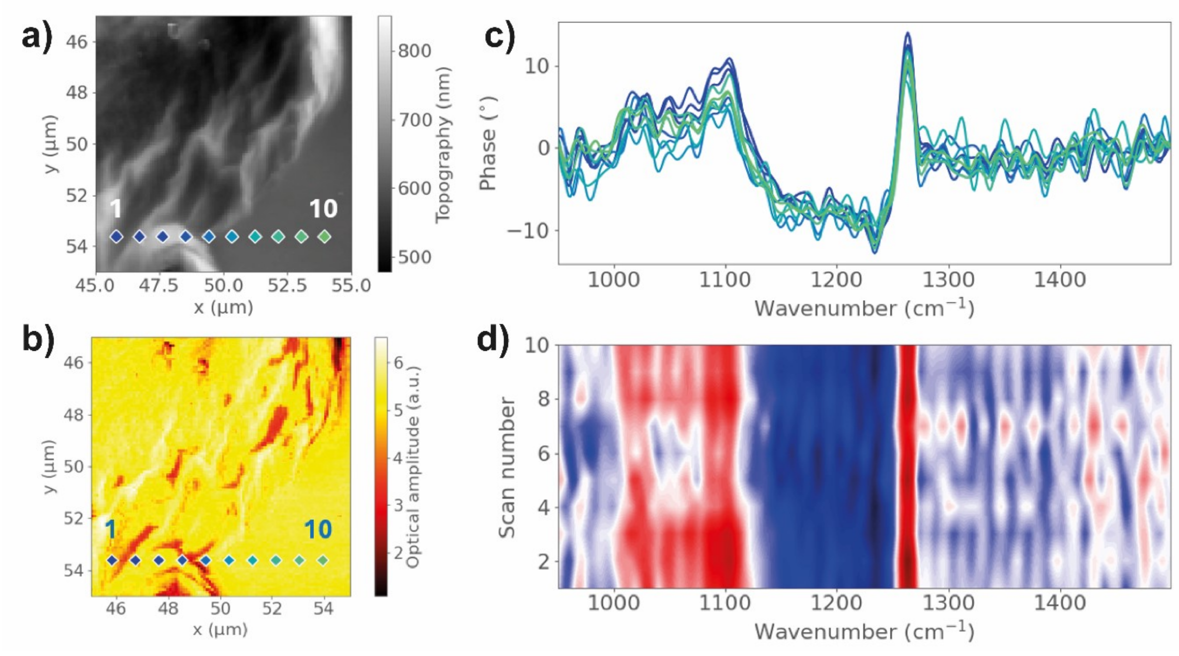

Figure S8. (a) AFM height topography and (b) its corresponding infrared absorption image of a large-size PDMS residue (>10 μm) transferred to the ITO surface. (c) Nano-FTIR spectra correspond to the points on a line marked in the AFM image. (d) The contour plot of nano-FTIR spectra corresponds to the line scan.

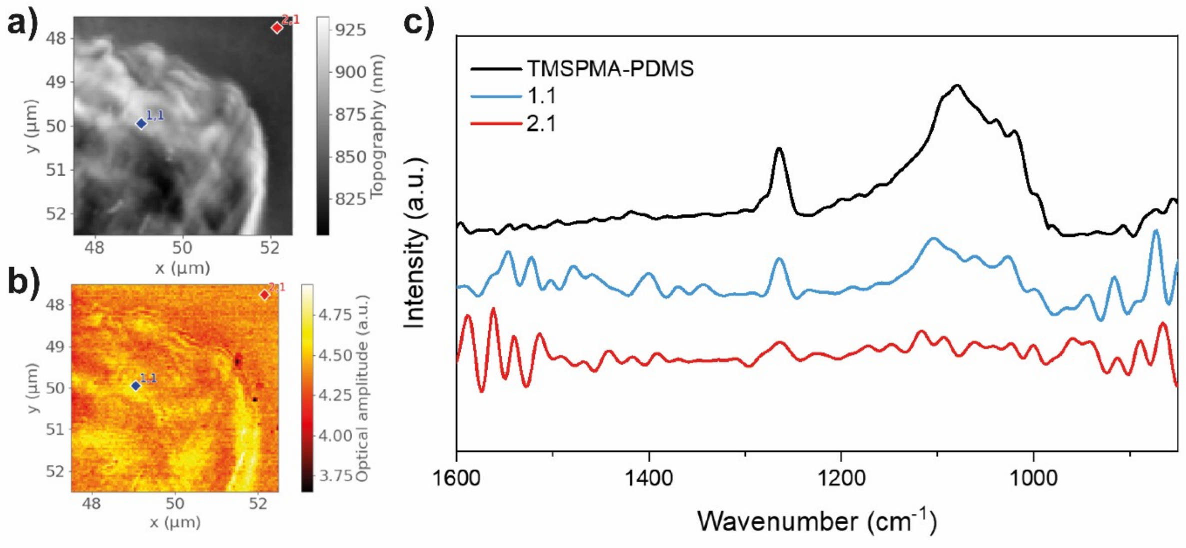

Figure S9. (a) AFM height topography and (b) its corresponding infrared absorption image of a medium-size TMSPMA-PDMS residue (<10 µm) transferred to the ITO surface. (c) Comparison of nano-FTIR spectra corresponds to the points marked on the AFM image and the neat TMSPMA-PDMS.

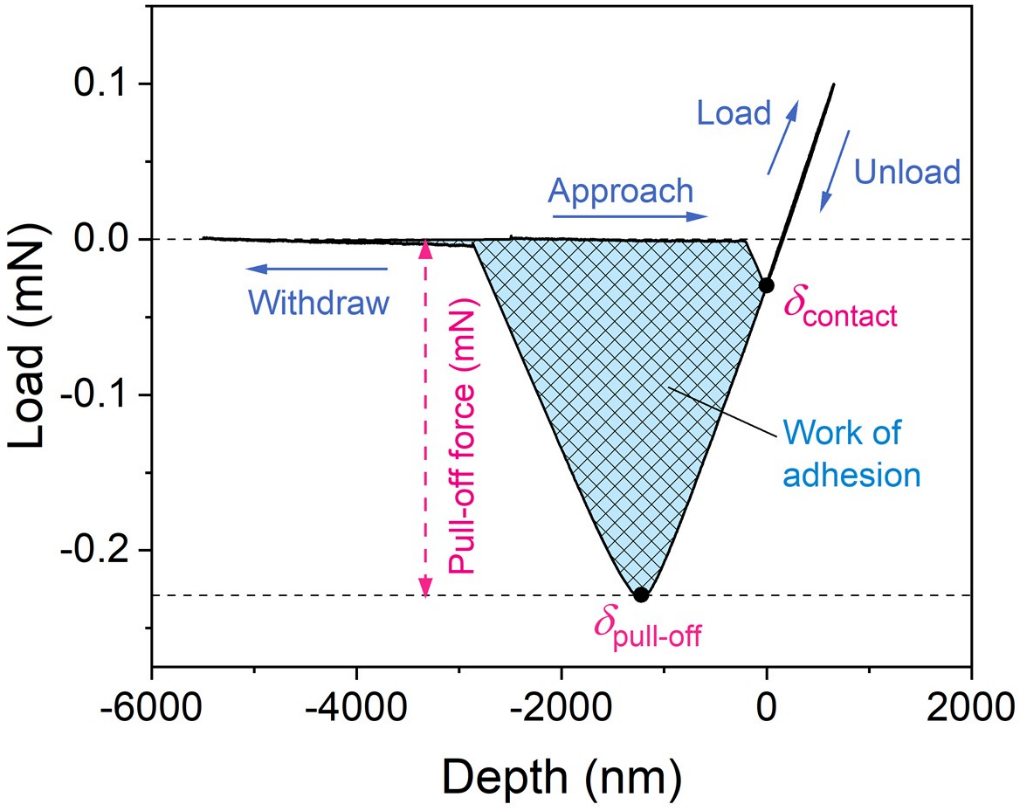

Figure S10. An example of load-depth curve obtained by a pull-off test conducted in an iMicro nanoindenter. $\delta_{contact}$ is the depth of indentation at the jump-to-contact point, and $\delta_{pull\text{-}off}$ is the indentation depth at maximum adhesive force.

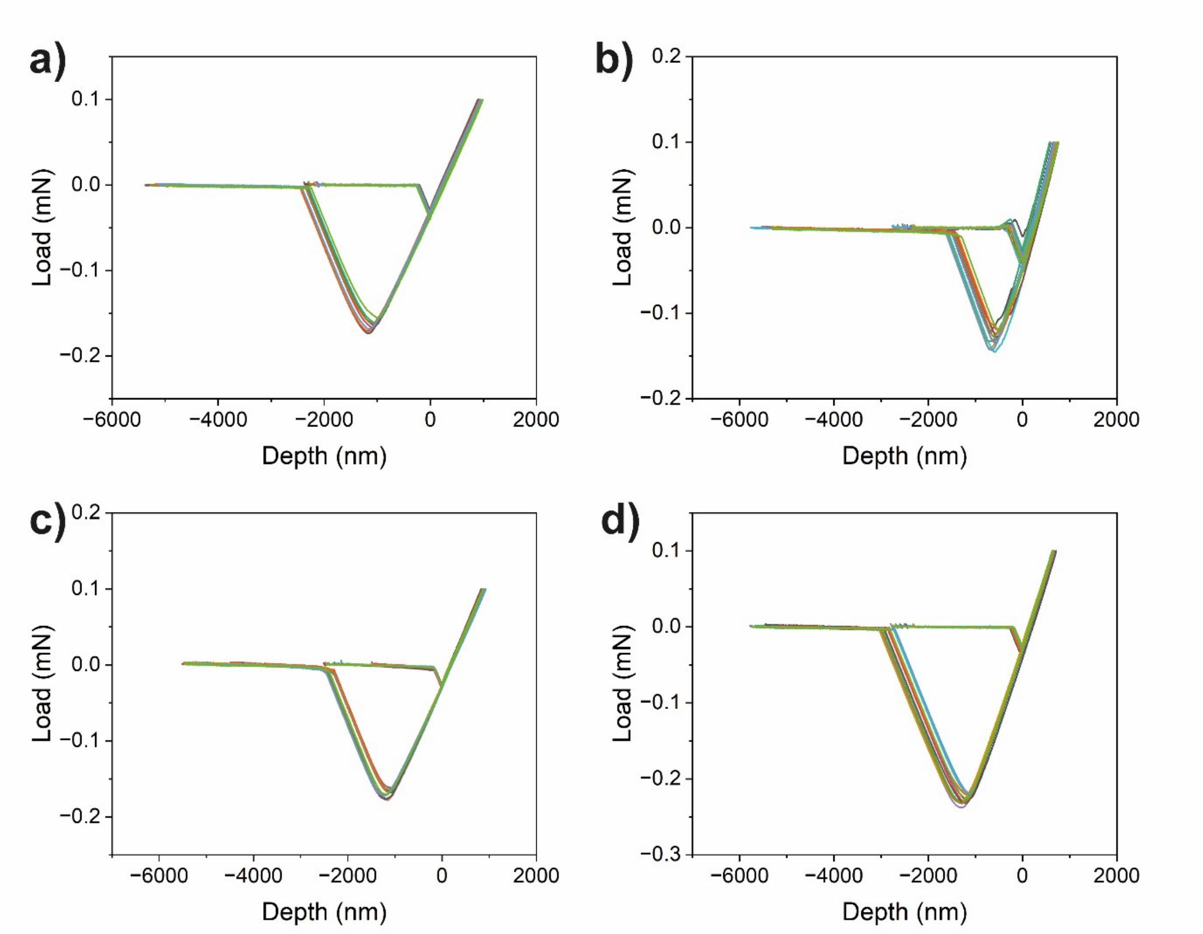

Figure S11. Load-depth curves of the PDMS samples obtained from pull-off surface adhesion tests, for the (a) pristine PDMS, (b) APTES-PDMS, (c) VTMS-PDMS, and (d) TMAPMS-PDMS samples, respectively. Each sample was tested at 12 distinctive positions.

References

[1] D. Nečas, P. Klapetek, Gwyddion: an open-source software for SPM data analysis, Open Phys. 10 (2012) 181-188. https://doi:10.2478/s11534-011-0096-2.
